# Antimicrobial Activity of Xanthohumol and Its Selected Structural Analogues

**DOI:** 10.3390/molecules21050608

**Published:** 2016-05-11

**Authors:** Monika Stompor, Barbara Żarowska

**Affiliations:** 1 Department of Chemistry, University of Environmental and Life Sciences, Norwida 25, 50-375 Wrocław, Poland; 2 Centre for Innovative Research in Medical and Natural Sciences, Faculty of Medicine, University of Rzeszów, Warzywna 1a, 35-310 Rzeszów, Poland; 3 Department of Biotechnology and Food Microbiology, University of Environmental and Life Sciences, Chełmońskiego 37/41, 51-630 Wrocław, Poland; barbara.zarowska@up.wroc.pl

**Keywords:** antimicrobial activity, xanthohumol, chalconaringenin, naringenin, 4′-methoxychalcones, *S. aureus*, NMR-IR-UV spectra

## Abstract

The objective of this study was to evaluate the antimicrobial activity of structural analogues of xanthohumol **1**, a flavonoid compound found in hops (*Humulus lupulus*). The agar-diffusion method using filter paper disks was applied. Biological tests performed for selected strains of Gram-positive (*Staphylococcus aureus*) and Gram-negative (*Escherichia coli*) bacteria, fungi (*Alternaria* sp.), and yeasts (*Rhodotorula rubra*, *Candida albicans*) revealed that compounds with at least one hydroxyl group—all of them have it at the C-4 position—demonstrated good activity. Our research showed that the strain *S. aureus* was more sensitive to chalcones than to the isomers in which the heterocyclic ring C is closed (flavanones). The strain *R. rubra* was moderately sensitive to only one compound: 4-hydroxy-4’-methoxychalcone **8**. Loss of the hydroxyl group in the B-ring of 4’-methoxychalcones or its replacement by a halogen atom (−Cl, −Br), nitro group (−NO_2_), ethoxy group (−OCH_2_CH_3_), or aliphatic substituent (−CH_3_, −CH_2_CH_3_) resulted in the loss of antimicrobial activity towards both *R. rubra* yeast and *S. aureus* bacteria. Xanthohumol **1**, naringenin **5**, and chalconaringenin **7** inhibited growth of *S. aureus*, whereas 4-hydroxy-4′-methoxychalcone **8** was active towards two strains: *S. aureus* and *R. rubra*.

## 1. Introduction

There is a growing interest in chemical compounds of natural origin having antimicrobial properties, which gives rise to a number of studies in this field. Current investigations into the antimicrobial activity of flavonoid compounds have targeted plant extracts, pure compounds, products of their metabolism, and also their synthetic analogues [1]. Nowadays chalcones which are precursors of all groups of flavonoid compounds, are more often recommended to combat different kinds of intestinal parasites that cause food poisoning in humans, for example *Giardia lamblia* [2]. They are also used as acetylcholinesterase inhibitors in the treatment of neurodegenerative diseases such as Alzheimer’s [3]. Additionally, chalcones demonstrate cytotoxic activity towards human cancer cells [4].

**Figure 1:**
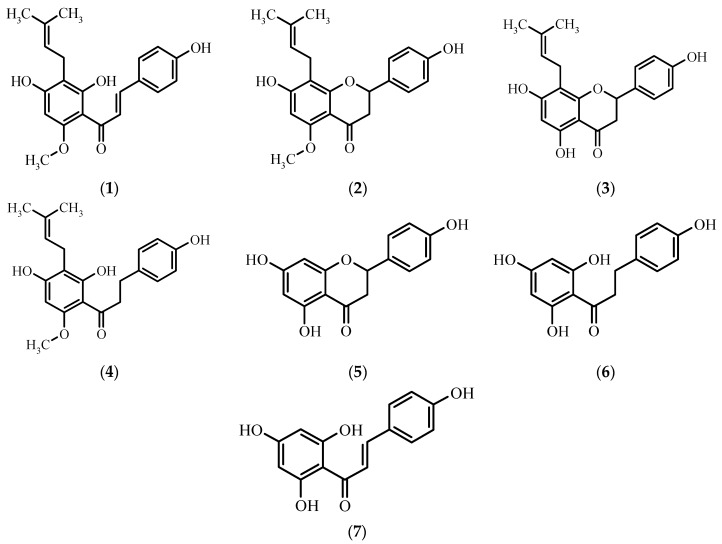
Chemical structures of xanthohumol **1** and its derivatives: isoxanthohumol **2**, 8-prenylnaringenin **3**, α,β-dihydroxanthohumol **4**, naringenin **5**, α,β-dihydrochalconaringenin **6**, and chalconaringenin **7**.

A valuable source of flavonoids and phenolic acids is beer, which is commonly consumed in the majority of countries all over the world [5]. Analysis of different brands of beer of Czech, Slovakian, Romanian, and Serbian origin revealed that it contains genistein, biochanin, daidzein, formononetin, quercetin, and apigenin [6,7]. Moreover, due to the addition of hops, beer contains such flavonoids as xanthohumol **1**, isoxanthohumol **2**, 8-prenylnaringenin **3**, trace amounts of α,β-dihydroxanthohumol **4** [8], naringenin **5**, and their derivatives (Figure 1).

In Poland, the best source of **1** in the diet is dark beer and light non-filtered beer, in which the content of this prenylated chalcone is estimated at up to 0.22 mg/L and is dependent on the amount of hop extract [9]. Germany has developed a technology for production of beer enriched in xanthohumol (“XAN technology”). It allows xanthohumol **1** concentratiosn of 1–3 mg/L in non-filtered lager-type beer and over 10 mg/L in filtered dark beer. In the Czech Republic “ŻATEC Xantho” beer contains 0.3 mg/L of **1** [10]. Encapsulated forms of hop extracts with estrogenic activity, rich in flavonoids, are known in Western Europe under the brand names Xantoflav™ (Germany), Lifenol, Naturex, and Avignon (France) [11]. Aromatic oils and bitter hop acids give beer its characteristic aroma and bitter taste and play a protective role against microbial infections [6,7,12]. The most recent reports suggest that there is a possibility of application of hop extract, containing bitter hop acids and **1**, in the meat industry as a natural preservative that inhibits the development of golden staph (*Staphylococcus aureus*) and thus increases food safety [13]. In addition, the antifeedant activity of hop compounds, selected synthetic chalcone derivatives, and chromones against the peach-potato aphid (*Myzus persicae*) has been reported recently [14].

Jayasinghe *et al.* [15] proved that plant geranylchalcones isolated from *Artocarpus nobilis* (*Moraceae*) have antifungal activity towards *Cladosporium cladosporioides*. Lόpez *et al.* [16] suggested that electron-donating substituents (e.g., −OCH_3_) in ring B of chalcones decrease their antifungal activity towards human dermatophytes, while electron acceptor groups (e.g., −NO_2_) at the *para* position have the opposite effect and augment this activity.

Isolated from *Dorstenia barteri* (*Moraceae*), isobavachalcone, which has a structure similar to the prenylated hop chalcone **1**, inhibited the growth of several pathogenic strains, including bacteria (IC_50_ = 0.3 μM for *Enterobacter cloacae*, *S. aureus*, *Bacillus stearothermophilus*), filamentous fungi, and yeast (IC_50_ = 0.3 μM for *C. albicans*, *Candida gabrata*). Significant results were obtained for two strains, *Microsporum audorium* and *Trichophyton rubrum*, where the measured IC_50_ value was the same as for nystatin, used as a reference standard [17].

Flavonoids may indirectly chelate transition metal ions, for example copper and iron, which prevents formation of the highly reactive hydroxyl radical in cells. Kaveri *et al.* [18] obtained ruthenium complexes with 2′-hydroxychalcone derivatives, substituted in the *para* position with a methyl group, chlorine, and naphthalene. The compounds were tested for antimicrobial activity against *S. aureus* and *Salmonella enterica*. The ruthenium compounds were more active than 2′-hydroxychalcone itself. Higher activity of the chelates was rationalized by the loss of the polar nature of the metal ion, caused by the delocalization of *π* electrons in the ring. Additionally, the lipophilic character of the whole compound increased, which resulted in better permeability of the conjugates through a phospholipid bilayer membrane of bacterial cells.

**Figure 2:**
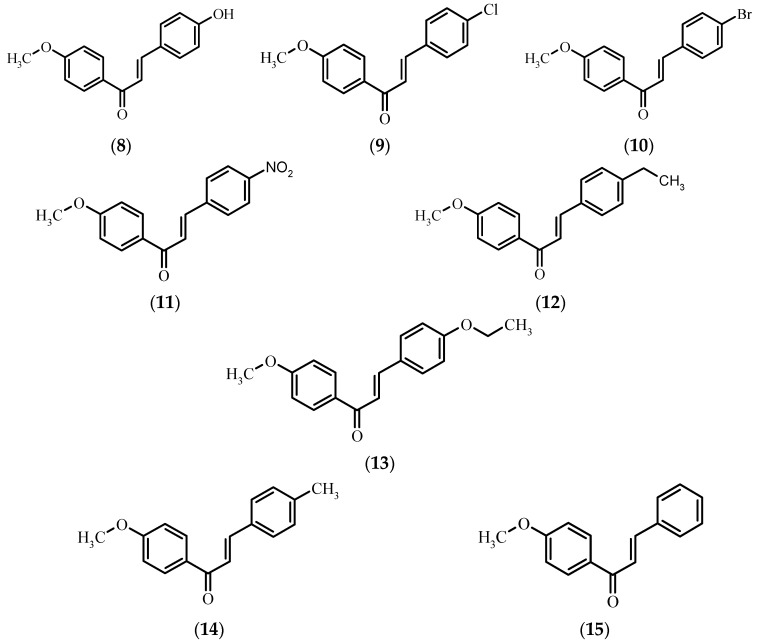
Chemical structure of 4′-methoxychalcones **8**–**15**.

Because of the constant risk of pathogenic bacterial infections, to assess the potential utility of the obtained compounds as antibacterial and antifungal agents we performed biological tests of xanthohumol **1**, naringenin **5**, chalconaringenin **7**, and eight 4′-methoxychalcones **8**–**15** for their antimicrobial activity against five microbial strains. The considerably low number of strains tested for microbicidal sensitivity to 4′-methoxychalcones (Figure 2) and the lack of information about any kind of biological activity of 4-bromo-4′-methoxychalcone **10** and 4-ethyl-4′-methoxychalcone **13** prompted us to undertake research in this field. To the best of our knowledge, there is no information about the antimicrobial activity of the rest of the compounds against the chosen microorganisms.

## 2. Results

### Antimicrobial Activity

For the biological tests we chose three hop flavonoids: xanthohumol **1**, chalconaringenin **7**, and naringenin **5**. Additionally, we tested a group of eight 4′-methoxychalcones **8**–**15**, differing in the electronegativity of the substituents at C-4. The minimal concentration that inhibits growth of a microorganism (MIC) for 4′-methoxychalcone **15**, known from the literature, determined towards ten different microorganisms was equal or more than 100 µg/mL [19]. For our tests we decided to use the disc diffusion method and to add to the group of microorganisms described in that work two strains that were not tested: *Alternaria* sp. KK-1 and *R. rubra* C-9. Additionally, we planned to check the impact of different substituents in the *para* position of the B aromatic ring of 4′-methoxychalcone **15** on the antimicrobial properties.

Initially, for assessment of the antimicrobial activity we planned to use the turbidimetric method with the help of the Bioscreen C system. Unfortunately, we observed that when a DMSO solution of a tested compound was added to the cultivation medium, a precipitate appeared. For this reason we used the agar disc diffusion method. In the case of substances that are poorly water-soluble, the drawback of this method is weak diffusion of the compounds into the agar medium, which was prepared in water [20,21]. For this reason we decided to use high concentrations of the tested compounds (**1**, **5**, **7**, **8**–**15**). However, despite such high doses, we observed that only four of the tested compounds demonstrated moderate antimicrobial activity, toward only one out of five tested strains. Some of the strains were totally resistant to the tested agents. Among the tested microorganisms only *S. aureus* PCM 2054 bacterium, and *R. rubra* C-9, yeast, were sensitive to the tested chalcones (Table 1).

**Figure 3:**
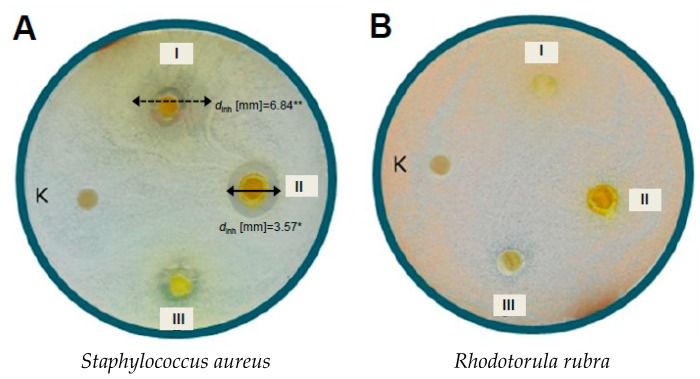
Influence of selected hydroxychalcones on the growth of the strains: (**A**) *S. aureus*, (**B**) *R. rubra*: (I) chalconaringenin (**7**); (II) xanthohumol (**1**); (III) 4-hydroxy-4′-methoxychalcone (**8**); (K) control (DMSO).

Among eight 4′-methoxychalcones **8**–**15** tested for antimicrobial activity, only 4-hydroxy-4′-methoxychalcone **8** inhibited the growth of the golden staph *S. aureus* and yeast *R. rubra* (Figure 3). The lack of hydroxyl group or its replacement by a halogen atom (−Cl **9**, −Br **10**), nitro group (−NO_2_ **11**), ethoxy group (−O−CH_2_CH_3_ **13**), or aliphatic groups (−CH_2_CH_3_ **12**), (−CH_3_ **14**) led to inactivation of the compounds with respect to both *R. rubra* yeast and *S. aureus* bacterium.

Studies on the relationship between compound structure and its activity towards bacteria revealed that the strain *S. aureus* is more sensitive to chalcones, compared to the isomers with a closed heterocyclic ring C. Chalconaringenin **7** and (±)-naringenin **5** considerably inhibited the growth of *S. aureus*, which resulted in the appearance of lighter zones around the discs with tested compounds (with much smaller number of microcolonies) of diameters *d*_inh_ = 6.84 mm and 3.77 mm, respectively. The obtained prenylated hop chalcone-xanthohumol **1** generated the largest inhibition zone of all tested chalcones in the case of the strain *S. aureus* (*d*_inh_ = 3.57 mm).

## 3. Discussion

The dilution method used by Rozalski *et al.* [22] in research on **1** with respect to three strains of *S. aureus* of different origin led to determination of the range of minimal inhibition concentration (MIC) as 15.6 to 62.5 µg/mL. Our results are consistent with the results of Ávila *et al.* [23], concerning evaluation of antimicrobial activity of xanthohumol **1** derivatives: isocordoin (which lacks 4-OH and C-6’-OCH_3_ substituents, compared to **1**) and 4-hydroxyisocordoin (lacking C-6′-OCH_3_ group). They proved that the presence of a hydroxyl group at C-4 is crucial for the antimicrobial activity towards *S. aureus*. Moreover, replacement of the substituent at C-8 in 4‑hydroxyisocordoin with a geranyl one did not affect the activity towards *S. aureus*, whereas growth of *Bacillus cereus* was more effectively inhibited by the derivative with the geranyl group. On the other hand, cyclization of the isoprenyl group resulted in a loss of activity. A few years later the observation that the electron-donating group in the B ring of chalcones has a positive effect on their antimicrobial activity was also confirmed by Wu *et al.* [24]. Similarly to our results, Gram-positive bacteria (*S. aureus*, *Staphylococcus epidermidis*, *Enterococcus faecalis*, *Enetrococcus faecium*) were more resistant to 4-hydroxy-4′-methoxychalcone **8** than Gram-negative ones (*E. coli*, *Klebsiella pneumonia*, *Pseudomonas aeruginosa*). Probably these differences are related to differences in composition of the cytoplasmic membrane in Gram-positive and Gram-negative bacteria. Similar assumptions were made in the research on the influence of hop extract on biochemical processes in Gram-positive and Gram-negative bacteria [25]. Karaman *et al.* [26] proved that substitution of 4′-methoxychalcone **15** at the *para* position in ring B with a chlorine atom results in deactivation of this compound towards the following pathogens: *Proteus vulgaris* KUEN 1329, *Candida utilis* KUEN 1031, and *P. aeruginosa* ATCC 9027. The activity of 4’-methoxychalcone **15** and 4-chloro-4′-methoxychalcone **9** towards *Streptomyces pyogenes* ATCC 176 was comparable—the diameters of inhibition zones were 9 and 10 mm, respectively. Conversely, a positive influence of the chlorine atom in **9** on the antimicrobial activity was observed for the anaerobic streptococcus *E. faecalis* ATCC 29122 (*d*_inh_ = 10 mm), which was resistant to non-substituted 4′-methoxychalcone **15**.

Our study showed that in the case of the second hop flavonoid, chalconaringenin **7** and 4-hydroxy-4′-methoxychalcone **8**, the zones of inhibition for *S. aureus* bacteria were smaller compared to xanthohumol **1**: 1.92 mm and 2.86 mm, respectively. The research on the structure–activity relationship in chalcone-flavone compounds with respect to bacterial sensitivity to antimicrobials showed that the strain *S. aureus* is more sensitive to chalcones than to the isomers in which the heterocyclic ring C is closed. Our research showed that this statement is also valid in the case of naringenin **5**, a flavonoid that is commonly found in citrus fruits, and its isomer, chalconaringenin **7**. Not only did these compounds considerably inhibit the growth of golden staph, which resulted in a lighter zone with a much lower number of visible microcolonies, but also chalcone form **7** was more active than the cyclic one, **5** (*d*_inh_ = 6.84 mm and 3.77 mm, respectively).

As the results show, the concentration of tested substances with respect to their growth inhibition activity is an important and so far not fully resolved issue. Our results add to the earlier study on antibiotic activity of naringenin **5** towards *S. aureus*, reported by Sasaki *et al.* [27], which showed that application of this flavonoid at a concentration of 250 µg/mL was not sufficient for inhibiting the growth of the golden staph bacterium. In the case of such strains as *Salmonella enteritidis* and *B. cereus*, the higher concentration of 400 µg/mL was also insufficient [28]. By contrast, the microbicidal activity of **5** towards Gram-negative *Acinetocabter haemolyticus* bacteria (MIC = 0.183 mmol/mL) was described by Chatzopoulou *et al.* [29]. Other strains tested by this group, including *Empedobacter brevis*, *P. aeruginosa*, and *K. pneumonia*, were resistant to **5**.

On the other hand, Shakil *et al.* [30], when comparing the microbial activity of chalcones and their isomers (flavanones) came to the conclusion that in the case of microorganisms other than *S. aureus* more active were compounds with the closed heterocyclic ring C. Only in the case of the non-substituted flavanone and 2’‑hydroxychalcone, and also for 4-bromoflavanone and 4-bromo-2′-hydroxychalcone, was there no difference in activity toward one of the two tested phytopathogens (*Rhizoctonia solani* ITCC 5563 and *Sclerotium rolfsii* ITCC 6181). The results for *R. solani* were similar for the two isomeric forms. This indicates that generalizations concerning the structure-activity relationship of the mentioned compounds cannot be applied to a wide range of microorganisms. The antibacterial activity of hydroxylated flavanone derivatives, including naringenin **5** towards streptococci *E. faecalis*, which is the bacterium most often found in hospitals (where there is usually a high level of resistance to antibiotics), was also confirmed by the other research group [31].

Moreover, the antimicrobial activity of the tested compounds may be dependent on the enzymatic system of the microorganisms and possible processes of detoxification. Degradation of **5** with the help of a chalcone isomerase of bacterial origin had been earlier described by Herles *et al.* [32]. This enzyme was isolated from the cells of anaerobic bacteria *Eubacterium ramulus* DSM 16296. In the first stage of conversion naringenin **5** underwent isomerization to chalconaringenin **7** and then, due to hydrogenation of the olefinic bond in the presence of catalytic amounts of NADH–α,β-dihydrochalconaringenin, **6** was formed. Chalconaringenin **7** also undergoes biotransformation to phloretin **6** in the cells of aerobic bacteria, such as *Rhodococcus* and *Gordonia*, as we described earlier [33]. The newest study proved the strong antibacterial properties of phloretin **6** and its glycoside derivatives towards Gram-positive (*S. aureus* ATCC 6538, *Listeria monocytogenes* ATCC 13932) and Gram-negative (*Salmonella typhimuriu*m ATCC 133111) bacteria [34]. As we reported earlier [35], when **1** was subjected to transformation by *Rhodotorula marina* AM 77, we obtained α,β-dihydroxanthohumol **4**. Most of the metabolites obtained in the biotransformations of **1**, including **4**, have not been tested for antimicrobial activity so far.

Our results showed that the strain *R. rubra* was moderately sensitive to only one compound: 4-hydroxy-4’-methoxychalcone **8**, which generated a lighter zone of inhibition (with a smaller number of microcolonies) of 2.46 mm diameter. Loss of the hydroxyl group or its replacement by a halogen atom (−Cl **9**, −Br **10**), nitro group (–NO_2_ **11**), ethoxy group (−OCH_2_CH_3_ **12**), or aliphatic substituent (−CH_3_ **14**), −CH_2_CH_3_ **13**) resulted in the loss of antimicrobial activity towards both *R. rubra* yeast and *S. aureus* bacterium. This is in agreement with the theory put forth by Batovska *et al.* [1], which states that the antifungal activity of chalcones depends on the presence of substituents that can easily enter into a Michael reaction with intracellular thiol groups. In this reaction the addition of thiols to the electrophilic center (C-β) of unsaturated compounds takes place.

## 4. Materials and Methods 

Reagents were purchased from Sigma-Aldrich (St. Louis, MO, USA) or Merck (Darmstadt, Germany). The commercially available (±)-naringenin **5** (Sigma-Aldrich) was used. The purity of the synthesized products (>95%) was established by HPLC chromatography. Analysis were performed according to the procedure described earlier [33]. The NMR spectra (^1^H-, ^13^C-NMR, ^13^C-DEPT, and correlation spectra: ^1^H-^1^H-COSY, ^1^H-^13^C-HMQC, ^1^H-^13^C-HMBC) were recorded in a CDCl_3_ solution on a Brüker Avance DRX 300 and Brüker Avance II 600 MHz spectrometers (Brüker, Karlsruhe, Germany). IR spectra (KBr discs) were determined on a Thermo-Nicolet IR300 FT-IR-spectrometer (Madison, WI, USA). UV spectra were run on a Visible Spectrofotometer Cintra 303, GBC, in methanol. Melting points (uncorrected) were determined on a Boetius apparatus. Bioscreen C (Lab system Oy, Helsinki, Finland) has been used in the initial part of the investigations of antimicrobial activity.

### 4.1. Antimicrobial Agents

#### 4.1.1. Xanthohumol and Chalconaringenin

Xanthohumol **1** was isolated from supercritical carbon-dioxide-extracted hops—a waste residue remaining after extraction of hops (*H. lupulus*) with supercritical CO_2_—received from the Supercritical Extraction Department of New Chemical Syntheses Institute in Puławy. Xanthohumol was obtained according to the method described earlier [36]. Spectral data of **1** were consistent with the literature [35]. The next hop chalcone and a precursor of the prenyl derivatives–chalconaringenin **7** was obtained by alkaline isomerization of racemic (±)-naringenin **5**, with the help of a methanolic KOH solution, according to the modified method of Le Bail *et al.* [37]. Spectral data of **7** were identical with literature values [33].

#### 4.1.2. (*E*)-4′-Methoxy-4-Substituted Chalcones

To 4′-methoxyacetophenone (12 mmol) dissolved in MeOH (10 mL) a benzaldehyde derivative (12 mmol) dissolved in MeOH (10 mL) was added portionwise. The reaction mixture was stirred for 10 min at room temperature and then KOH (1.44 g, 36 mmol) was added. The resulting mixture was stirred for 14–18 h in the dark. Next the mixture was diluted with three volumes of distilled water and acidified to pH 4–6 with concentrated HCl. The precipitate was filtered on a Schott filter, washed with large amounts of distilled water, and dried under a vacuum for 5 h. The crude product was purified by column chromatography on silica gel (eluent: 10%–20% *v*/*v* of acetone in hexane). A series of respective (*E*)-1,3-diphenylpropen-1-one derivatives was obtained in 60%–85% yield (see Supplementary Materials).

By this method we obtained 4′-methoxychalcone **15** and its seven 4′-methoxy *para-*substituted derivatives **8**–**14**. In order to introduce substituents of a different chemical nature at the C-4 position, as substrates we used 4-methoxyacetophenone and benzaldehyde or its derivatives, as follows: 4-chlorobenzaldehyde, 4-bromobenzaldehyde, 4-hydroxybenzaldehyde, 4-nitrobenzaldehyde, 4-methylbenzaldehyde 4-ethylbenzaldehyde, and 4-ethoxybenzaldehyde. The products obtained were purified and characterized by means of spectral methods (NMR, IR) and spectrophotometric analysis (UV).

### 4.2. Evaluation of Antimicrobial Activity

Evaluation of antimicrobial activity of selected compounds **1**, **5**, **7**, and **8**–**15** were performed against the strains of bacteria (*Escherichia coli* PCM 2560 and *S. aureus* PCM 2054), yeasts (*C. albicans* KL-1 and *Rhodotorula rubra* C-9), and filamentous fungi (*Alternaria* sp. KK-1). The analyses were carried out by the agar diffusion method using filter paper disks. For the study we used the microorganisms from the collection of pure cultures of the Department of Biotechnology and Food Microbiology at Wrocław University of Environmental and Life Sciences and from the Polish Collection of Microorganisms (PCM), Ludwik Hirszfeld Institute of Immunology and Experimental Therapy of the Polish Academy of Sciences, Wrocław, Poland.

YM agar (for yeasts and fungi) and nutrient broth (for bacteria) were inoculated with a cell suspension of the selected microorganisms (standardized with a Thoma chamber for yeasts or with McFarland standards for bacteria) to give a final concentration of 5 × 10^5^ CFU/mL. On the surface of the plates prepared in this way sterile filter-paper discs of 0.5 cm diameter were placed and then portions of 10 µL of 20% (*m*/*v*) solution of a tested compound in DMSO were transferred onto the discs. At the same time control samples were prepared in an analogous manner, but in this case only DMSO was applied on the discs. As reference standards we used oxytetracycline (for bacteria) and cycloheximide (for yeasts and filamentous fungi), which were added at the same concentration as the tested compounds. The plates were left for 8 h at 4 °C to enable diffusion of the tested compounds to the cultivation medium, and then incubated at 25 °C (filamentous fungi) or at 30 °C (yeasts and bacteria) for 48–96 h. A clear zone of inhibition with no microcolonies observed around the disc of a tested compound was interpreted as its ability to inhibit growth of the microorganism inoculated in the medium. A visibly lighter zone of inhibition, with a smaller number of microcolonies, was interpreted as the capability to partially inhibit the growth of a tested strain.

## 5. Conclusions

The aim of the study was to search for antimicrobial compounds in the group of natural flavonoids contained in hops (*Humulus lupulus*) and their synthetic derivatives. The 4’-methoxychalcones (**8**–**15**) were obtained in order to evaluate the impact of compound structures, including electron donating and electron acceptor substituents at *para* position in ring B on antimicrobial activity of these compounds. 

The strain *S. aureus* was sensitive toward four out of eleven tested compounds. These compounds contain at least one hydroxyl group (**1**, **5**, **7**, **8**), and all of them have it at C-4 position. The most active compound towards tested Gram-positive bacteria was xanthohumol (**1**), for which inhibition zone diameter in the case of *S. aureus* was *d*_inh_ = 3.57 mm. The strain *R. rubra* was sensitive to only one compound: 4-hydroxy-4’-methoxychalcone (**8**).

## Figures and Tables

**Table 1:** Antimicrobial activity of tested compounds.

Compound Number	Microorganism				
	*E. coli*	*S. aureus*	*C. albicans*	*R. rubra*	*Alternaria* sp.
	*d*_inh_ (mm)				
(**1**)	-	3.57 *	-	-	-
(**5**)	-	3.77 **	-	-	-
(**7**)	-	1.92 ** 6.84 **	-	-	-
(**8**)	-	2.86 *	-	2.46 **	-
(**9**)	-	-	-	-	-
(**10**)	-	-	-	-	-
(**11**)	-	-	-	-	-
(**12**)	-	-	-	-	-
(**13**)	-	-	-	-	-
(**14**)	-	-	-	-	-
(**15**)	-	-	-	-	-
oxytetracycline	4.17 *	9.18 *			
cyklohexymide			13.21 *	11.32 *	3.91 *

* Zone of inhibition in mm; ** lighter zone of inhibition in mm; “-“no activity.

## Supplementary Materials

Supplementary materials can be accessed at: http://www.mdpi.com/1420-3049/21/5/608/s1.
