# Supplementary material for: Antimicrobial Activity of Xanthohumol and Its Selected Structural Analogues
Source: Molecules. 2016 May 11;21(5):608. doi: 10.3390/molecules21050608 (PMC6272900; doi:10.3390/molecules21050608)
Supplement: Supplementary file 1 [file molecules-21-00608-s001.pdf]

Supplementary Materials: Antimicrobial Activity of Xanthohumol and Its Selected Structural Analogues

Monika Stompor and Barbara Żarowska

4-Hydroxy-4-methoxychalcone (**8**) (1.9 g, yield = 65%) m.p. = 180–183 °C (MeOH). IR (KBr) cm^{-1}: 3182, 1643, 1601, 1579, 1514, 1439, 1343, 1263, 1228, 1166, 1023, 978, 831. ^{1}H-NMR (600 MHz, acetone-d_6) δ (ppm): 8.91 (1H, s, –OH), 8.13 (2H, d, *J* = 8.8 Hz, H-2′, H-6′, AA′BB′), 8.00 (1H, d, *J* = 15.5 Hz, H-α), 7.72 (1H, d, *J* = 15.5 Hz, H-β), 7.70 (2H, d, *J* = 8.6 Hz, H-2, H-6, AA′BB′), 7.06 (2H, d, *J* = 8.8 Hz, H-3′, H-5′, AA′BB′), 6.92 (2H, d, *J* = 8.6 Hz, H-3, H-5, AA′BB′), 3.90 (3H, s, –OCH_3). UV (MeOH) λ_{max}: 347.4; 234.1 nm.

4-Chloro-4′-methoxychalcone (**9**) (2.5 g, yield = 78%) m.p. = 130–131 °C (MeOH). IR (KBr) cm^{-1}: 2962, 1656, 1605, 1510, 1492, 1330, 1254, 1223, 1177, 1092, 1024, 981, 818. ^{1}H-NMR (600 MHz, $CDCl_3$) δ (ppm): 8.03 (2H, d, *J* = 8.8 Hz, H-2′, H-6′, AA′BB′), 7.74 (1H, d, *J* = 15.6 Hz, H-α), 7.57 (2H, d, *J* = 8.5 Hz, H-2, H-6, AA′BB′), 7.51 (1H, d, *J* = 15.6 Hz, H-β), 7.38 (2H, d, *J* = 8.5 Hz, H-3, H-5, AA′BB′), 6.98 (2H, d, *J* = 8.8 Hz, H-3′, H-5′), 3.89 (3H, s, –OCH_3) ^{13}C-NMR (150 MHz, $CDCl_3$) δ: 188.4 (C=O), 163.5, 142.4, 136.2, 133.6, 130.9, 130.8, 129.5, 129.2, 122.3, 113.9, 55.5 (–OCH_3). UV (MeOH) λ_{max}: 318.6; 229.3 nm.

4-Bromo-4′-methoxychalcone (**10**) (2.8 g, yield = 75%) m.p. = 150–151 °C (MeOH). IR (KBr) cm^{-1}: 3014, 1657, 1603, 1577, 1560, 1510, 1486, 1331, 1314, 1257, 1223, 1176, 1036, 1021, 979, 815. ^{1}H-NMR (600 MHz, $CDCl_3$) δ (ppm): 8.03 (2H, d, *J* = 7.7 Hz H-2′, H-6′,), 7.72 (1H, d, *J* = 15.6 Hz, H-α), 7.55–7.49 (5H, m, H-3, H-5, H-2, H-6, H-β), 6.98 (2H, d, *J* = 7.7 Hz, H-3′, H-5′), 3.89 (3H, s, –OCH_3). ^{13}C-NMR (150 MHz, $CDCl_3$) δ: 188.4 (C=O), 163.5, 142.5, 134.0, 132.1, 130.9, 130.8, 129.7, 124.5, 122.4, 113.9, 55.5 (–OCH_3). UV (MeOH) λ_{max}: 320.5; 229.3 nm.

4′-Methoxy-4-nitrochalcone (**11**) (2.4 g, yield = 73%) m.p. = 170–172 °C (MeOH). IR (KBr) cm^{-1}: 2895, 1659, 1613, 1599, 1516, 1344, 1268, 1227, 1177, 1021, 826. ^{1}H-NMR (600 MHz, $CDCl_3$) δ (ppm): 8.27 (2H, d, *J* = 8.8 Hz H-2′, H-6′, AA′BB′), 8.05 (2H, d, *J* = 8.8 Hz, H-2, H-6, AA′BB′), 7.80 (1H, d, *J* = 15.7 Hz, H-α), 7.78 (2H, d, *J* = 8.8 Hz, H-3′, H-5′, AA′BB′), 7.65 (1H, d, *J* = 15.7 Hz, H-β), 7.00 (2H, d, *J* = 8.8 Hz, H-3, H-5, AA′BB′), 3.91 (3H, s, –OCH_3). ^{13}C-NMR (150 MHz, $CDCl_3$) δ: 187.7 (C=O), 163.9 (C-4′), 148.4 (C-1′), 141.3 (C-4), 140.7 (C-α), 131.0 (C-2, C-6), 130.5 (C-1), 128.8 (C-3′, C-5′), 125.6 (C-β), 124.2 (C-2′, C-6′), 114.0 (C-3, C-5), 55.6 (–OCH_3). UV (MeOH) λ_{max}: 323.4 nm.

4-Ethyl-4′-methoxychalcone (**12**) (2.7 g, yield = 85%) IR (KBr) cm^{-1}: 2967, 2933, 1655, 1603, 1569, 1510, 1421, 1333, 1315, 1260, 1223, 1175, 1113, 1032, 1015, 987, 826. ^{1}H-NMR (300 MHz, $CDCl_3$) δ (ppm): 8.07 (2H, d, *J* = 9.0 Hz, H-2′, H-6′, AA′BB′), 7.82 (1H, d, *J* = 15.6 Hz, H-α), 7.60 (2H, d, *J* = 8.1 Hz, H-2, H-6), 7.55 (1H, d, *J* = 15.6 Hz, H-β), 7.27 (2H, d, H-3, H-5), 7.01 (2H, d, *J* = 9.0 Hz, H-3′, H-5′, AA′BB′), 3.92 (3H, s, –OCH_3), 2.71 (2H, q, –CH_2), 1.28 (3H, t, *J* = 7.6 Hz, –CH_3).^{13}C-NMR (75 MHz, $CDCl_3$) δ (ppm): 188.8 (C=O), 163.3, 147.1, 144.0, 132.5, 131.2, 130.7, 128.5, 120.9, 113.8, 55.5 (–OCH_3), 28.8 (–CH_2) 15.3 (–CH_3). UV (MeOH) λ_{max}: 325.3; 226.4 nm.

4-Ethoxy-4′-methoxychalcone (**13**) (2.8 g, yield = 84%) m.p. = 105–106 °C (EtOH). IR (KBr) cm^{-1}: 3055, 2936, 1655, 1602, 1572, 1509, 1492, 1445, 1422, 1336, 1309, 1286, 1259, 1225, 1187, 1035, 1016, 1187, 973, 992, 830, 763. ^{1}H-NMR (300 MHz, $CDCl_3$) δ (ppm): 8.03 (2H, d, *J* = 8.9 Hz, H-2′, H-6′, AA′BB′), 7.78 (1H, d, *J* = 15.6 Hz, H-α), 7.59 (2H, d, *J* = 8.8 Hz, H-2, H-6, AA′BB′), 7.43 (1H, d, *J* = 15.6 Hz, H-β), 6.98 (2H, d, *J* = 8.9 Hz, H-3′, H-5′, AA′BB′), 6.92 (2H, d, *J* = 8.8 Hz, H-3, H-5, AA′BB′), 4.07 (2H, q, –CH_2), 3.88 (3H, s, –OCH_3), 1.44 (3H, t, *J* = 7.0 Hz, –CH_3). ^{13}C-NMR (75 MHz, $CDCl_3$) δ (ppm): 188.7 (C=O), 163.2, 160.9, 143.9, 131.4, 130.7, 130.1, 127.6, 119.4, 114.8, 113.7, 63.6 (–CH_2–), 55.4 (–OCH_3), 14.7 (–CH_3). UV (MeOH) λ_{max}: 344.5; 234.1 nm.

4′-Methoxy-4-methylchalcone (**14**) (2.4 g, yield = 80%) m.p. = 125–127 °C (MeOH). IR (KBr) cm^{-1}: 3010, 2954, 1654, 1599, 1571, 1509, 1418, 1332, 1308, 1255, 1226, 1175, 1034, 1018, 985, 839, 811.^{1}H-NMR (300 MHz, $CDCl_3$) δ (ppm): 8.04 (2H, d, *J* = 8.9 Hz, H-2′, H-6′, AA′BB′), 7.79 (1H, d, *J* = 15.6 Hz, H-α),

7.55 (2H, d, *J* = 8.0 Hz, H-2, H-6, AA'BB'), 7.51 (1H, d, *J* = 15.6 Hz, H-*β*), 7.22 (2H, d, *J* = 8.0 Hz, H-3, H-5, AA'BB'), 6.98 (2H, d, *J* = 8.9 Hz, H-3', H-5', AA'BB'), 3.89 (3H, s, –OCH_3), 2.39 (3H, s, –CH_3).^{13}C-NMR (75 MHz, $CDCl_3$) *δ* (ppm): 188.8 (C=O), 163.3, 144.0, 140.8, 132.3, 131.2, 130.7, 129.6, 128.4, 120.8, 113.8, 55.5 (–OCH_3) 21.5 (–CH_3).UV (MeOH) λ_{max}: 326.2; 231.2 nm.

4'-Methoxychalcone (**15**) (2.4 g, yield = 85%) m.p. = 107–109 °C (MeOH). IR (KBr) cm^{-1}: 3056, 1655, 1603, 1573, 1446, 1337, 1260, 1036, 974, 830, 763. ^{1}H-NMR (600 MHz, $CDCl_3$) *δ* (ppm): 8.04 (2H, d, *J* = 7.8 Hz, H-2', H-6'), 7.80 (1H, d, *J* = 15.6 Hz, H-*α*), 7.64 (2H, d, *J* = 7.04 Hz, H-2, H-6), 7.55 (1H, d, *J* = 15.6 Hz, H-β), 7.41–7.39 (3H, m, H-3, H-4, H-5), 6.99 (2H, d, *J* = 7.8 Hz, H-3', H-5'), 3.89 (3H, s, –OCH_3). ^{13}C-NMR (150 MHz, $CDCl_3$) *δ* (ppm): 188.7 (C=O), 163.4, 144.0, 135.1, 131.1, 130.8, 130.3, 128.9, 128.3, 121.9, 113.8, 55.5 (–OCH_3). UV (MeOH) λ_{max}: 316.6; 227.4 nm.
